# Novel model integrating computed tomography-based image markers with genetic markers for discriminating radiation pneumonitis in patients with unresectable stage III non-small cell lung cancer receiving radiotherapy: a retrospective multi-center radiogenomics study

**DOI:** 10.1186/s12885-023-11809-y

**Published:** 2024-01-15

**Authors:** Jiaran Li, Li Li, Shanshan Tang, Qingxi Yu, Wenju Liu, Ning Liu, Fengchang Yang, Dexian Zhang, Shuanghu Yuan

**Affiliations:** 1 Shandong University Cancer Center, Jinan, Shandong China; 2 Department of Radiation Oncology, Shandong Provincial Key Laboratory of Radiation Oncology, Shandong Cancer Hospital and Institute, Shandong First Medical University, Shandong Academy of Medical Sciences, Jinan, Shandong China; 3 Department of Radiation Oncology, The Affiliated Cancer Hospital of Zhengzhou University, Zhengzhou, Henan China; 4 Department of Radiation Oncology, Liaocheng Pepole’s Hospital, Liaocheng, Shandong China; 5 Department of Pathology, Shandong Cancer Hospital and Institute, Shandong First Medical University, Shandong Academy of Medical Sciences, Jinan, Shandong China

**Keywords:** Radiation pneumonitis, Lung cancer, Radiomics, Genomics, Radiogenomics, Prediction model

## Abstract

**Background:**

Chemoradiotherapy is a critical treatment for patients with locally advanced and unresectable non-small cell lung cancer (NSCLC), and it is essential to identify high-risk patients as early as possible owing to the high incidence of radiation pneumonitis (RP). Increasing attention is being paid to the effects of endogenous factors for RP. This study aimed to investigate the value of computed tomography (CT)-based radiomics combined with genomics in analyzing the risk of grade ≥ 2 RP in unresectable stage III NSCLC.

**Methods:**

In this retrospective multi-center observational study, 100 patients with unresectable stage III NSCLC who were treated with chemoradiotherapy were analyzed. Radiomics features of the entire lung were extracted from pre-radiotherapy CT images. The least absolute shrinkage and selection operator algorithm was used for optimal feature selection to calculate the Rad-score for predicting grade ≥ 2 RP. Genomic DNA was extracted from formalin-fixed paraffin-embedded pretreatment biopsy tissues. Univariate and multivariate logistic regression analyses were performed to identify predictors of RP for model development. The area under the receiver operating characteristic curve was used to evaluate the predictive capacity of the model. Statistical comparisons of the area under the curve values between different models were performed using the DeLong test. Calibration and decision curves were used to demonstrate discriminatory and clinical benefit ratios, respectively.

**Results:**

The Rad-score was constructed from nine radiomic features to predict grade ≥ 2 RP. Multivariate analysis demonstrated that histology, Rad-score, and XRCC1 (rs25487) allele mutation were independent high-risk factors correlated with RP. The area under the curve of the integrated model combining clinical factors, radiomics, and genomics was significantly higher than that of any single model (0.827 versus 0.594, 0.738, or 0.641). Calibration and decision curve analyses confirmed the satisfactory clinical feasibility and utility of the nomogram.

**Conclusion:**

Histology, Rad-score, and XRCC1 (rs25487) allele mutation could predict grade ≥ 2 RP in patients with locally advanced unresectable NSCLC after chemoradiotherapy, and the integrated model combining clinical factors, radiomics, and genomics demonstrated the best predictive efficacy.

## Introduction

Chemoradiotherapy plays a critical role in the management of patients with locally advanced and unresectable non-small cell lung cancer (NSCLC) [1]. Advances in the delivery of thoracic radiotherapy (RT) have the potential for local tumor control and improved prognosis in patients with NSCLC. However, these benefits come at the expense of an increased risk of radiation-induced lung toxicity (RILT) [2, 3]. Radiation pneumonitis (RP) is the major dose-limiting RILT for RT, and approximately 15−40% of patients with NSCLC experience symptomatic RP [4]. In severe cases, RP can lead to mortality rates as high as 1.9%, as reported in an international meta-analysis of individual patient data [5]. Traditionally, RP prediction is based on dosimetric parameters and clinical characteristics [6]. However, the current prediction models based on these factors have not yielded satisfactory results. Therefore, there is an urgent need for a novel predictive model that considers the individual heterogeneous responses to radiation. Such a model may help identify and prevent RP in high-risk patients before the onset of symptoms.

Computed tomography (CT) plays a pivotal role in diagnosis and treatment of RP. In recent years, with the rapid development of image-based radiomics analysis technology, there has been an increasing focus on using radiomics features to predict the effects of RT and adverse events, as they can provide additional information based on high-dimensional quantitation of medical images [7]. Du et al. successfully established a predictive model for RP by analyzing the region of interest (ROI) of whole lung tissue using cone-beam CT radiomics [8]. A nomogram model combining radiomics and clinical features showed superior predictive ability compared with other predictors. Given the increasing attention paid to endogenous factors and radiosensitivity in the context of radiation-related adverse effects [9], building predictive models solely from clinical and imaging perspectives may not be sufficient.

Microarray-based gene expression signatures are used in cancer diagnostics, tumor classification, prognosis, and the prediction of treatment responses [10, 11]. Gene expression status between individuals or tumors contributes significantly to differences in the occurrence of RP [12]. With the advent of genomic sequencing in the era of personalized medicine, it is imperative to explore multiple integrated approaches that incorporate genome-wide genotypic data to predict radiation-induced toxicity. However, there is a lack of studies investigating whether a model combining image biomarkers with genetic biomarkers can achieve superior RP identification in patients with lung cancer following RT.

In this study, we first aimed to evaluate the capability of CT-based radiomics to characterize RILT and determine whether our constructed radiomic features could be potential imaging markers for predicting RP. Additionally, we developed a comprehensive nomogram model incorporating radiomics features with gene expression alteration signatures for individualized risk assessment and precise prediction of RP.

## Methods

### Ethics statements

**Fig. 1:**
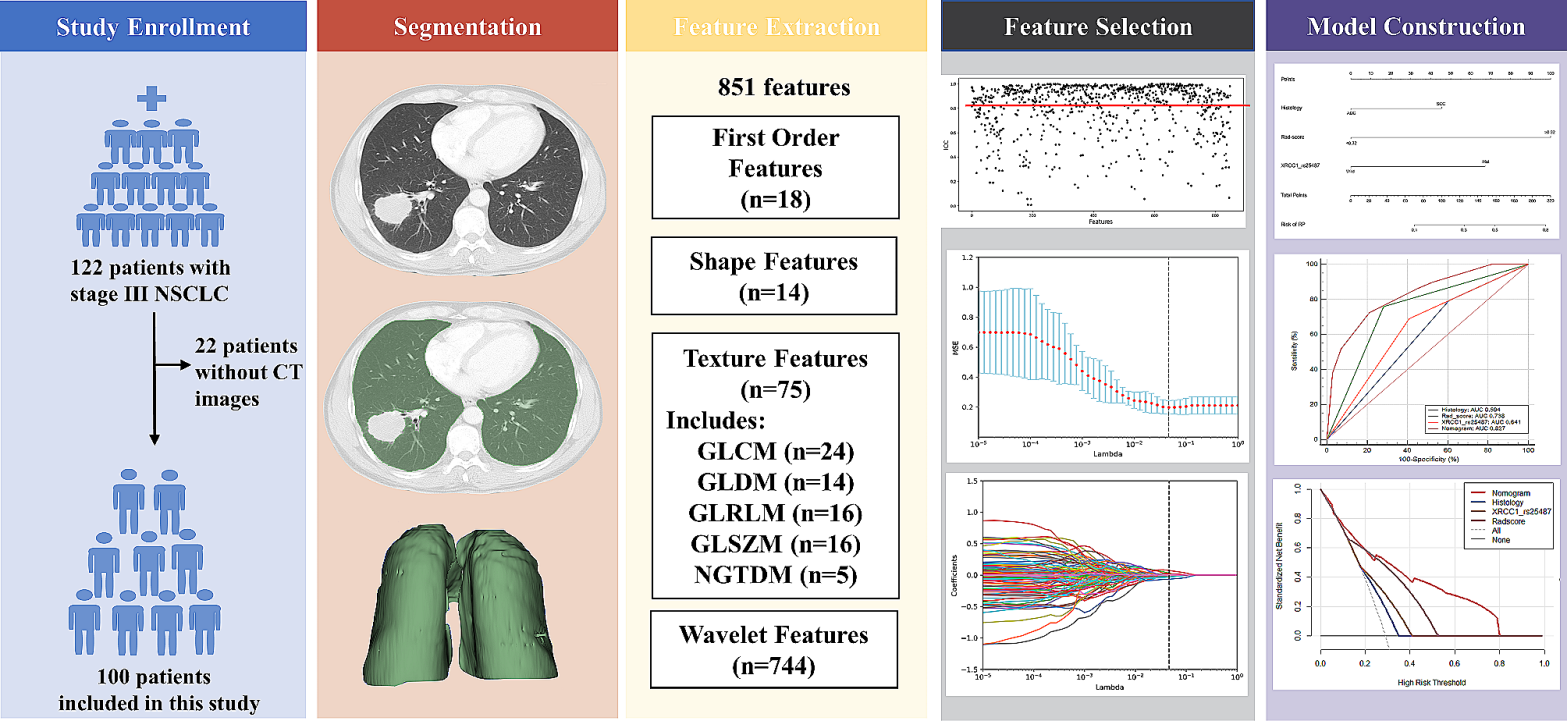
Workflow of the study

This study was approved by the Ethical Review Board of Shandong Cancer Hospital and Institute (ethics approval number: SDTHEC2020004042), and all patients provided written informed consent. The present study was conducted in compliance with the standard TRIPOD guidelines for prediction models. The workflow of this study is shown in Fig. 1.

### Study design and population

This retrospective study, which aimed to evaluate the capability of CT-based radiomics to characterize RILT and determine whether our constructed radiomic features could be potential imaging markers for predicting RP, included 100 patients with NSCLC who were treated with chemoradiotherapy at multiple centers between October 2014 and March 2019. Patients were eligible for this study based on the following criteria: histological diagnosis of unresectable stage IIIA-C NSCLC based on the 8th edition of AJCC TNM staging system without severe pleural or pericardial effusion; age >18 years; adequate lung, bone marrow, renal, hepatic, and cardiac function; and no history of systemic treatment or radiotherapy for thoracic cancers. CT images, gene panels, and the clinical characteristics of each patient were available.

### Treatment and evaluation of RP

All patients underwent standard definitive chemoradiotherapy (dCRT). A median of five cycles of cisplatin- or paclitaxel-based chemotherapy was administered sequentially or concurrently with RT. The choice of the chemotherapy regimen was left to the investigator’s discretion. Intensity-modulated radiation therapy or three-dimensional conformal radiation therapy was administered at a total dose of 50−70 Gy.

Follow-up visits were conducted 1 month after RT and every 3months during the first year. Subsequently, the patients were followed up every 3−6 months. RT-associated pneumonitis was graded according to the toxicity criteria of the Radiation Therapy Oncology Group and the European Organization for Research and Treatment of Cancer [13]. The primary outcome of RP was defined as symptomatic RP of ≥grade 2 within 6 months after RT. RP monitoring was based a combination of clinical symptoms, outpatient medical records, laboratory test results, and visual inspection of follow-up CT scans.

### CT image acquisition, segmentation and feature extraction

CT images in the Digital Imaging and Communications in Medicine format were extracted from the PACS system and then imported into 3D Slicer software (version 5.0.3; http://www.slicer.org) to extract and analyze radiomics features. The region of the entire (left plus right) lung, regarded as the ROI, was semi-automatically delineated and then manually modified in a slice-by-slice manner on lung-window CT baseline images by an experienced radiation oncologist. Tumors, diaphragms, trachea and mainstem bronchi were excluded. Another radiation oncologist independently reviewed lung organ segmentation, and any disputes were resolved by direct consultation between the two radiologists. All features were extracted using the radiomics plug-in of the 3D Slicer software.

### Radiomics feature selection and signature construction

To validate the reproducibility of the extracted features and minimize operator bias, 20 patients were randomly selected for repeated segmentation by an experienced radiologist at 2-month intervals from the initial evaluation to reacquire imaging features. Subsequently, the intraclass correlation coefficient (ICC) was calculated, and only features with an ICC of ≥ 0.8 were selected for further analysis. To avoid heterogeneity bias, normalization (z-score transformation) of the image intensity was performed on the entire image to transform the CT values into standardized intensity ranges.

The least absolute shrinkage and selection operator (LASSO) algorithm was used to determine the most predictive features. During the model building process,. the optimum parameter lambda (λ) was selected from the LASSO model using 10-fold cross validation with the minimum mean square error. With an increasing penalty, more regression coefficients are reduced to zero [14, 15], and the remaining non-zero coefficient is selected. After feature selection, a radiomics signature, also termed the Rad-score, was established from a linear combination of the selected features and the corresponding coefficients derived from the LASSO model.

### Gene mutation signatures

As our previously described [16], target sequencing of 474 cancer- and radiotherapy-related gene panels was performed on tumor tissue samples from each patient to identify genetic markers associated with the incidence of radiation-induced thoracic toxicity. Our result demonstrated that single nucleotide polymorphisms in XRCC5 (rs3835), XRCC1 (rs25487), MTHFR (rs1801133), and NQO1 (rs1800566) and somatic alterations in ZNF217 and POLD1 were associated with an increased risk of RP.

### Radiomics nomogram construction

The receiver operating characteristic (ROC) curve for the ability of the Rad-score to predict RP was plotted, and the point on the curve with the largest Youden index was selected as the cut-off value for the Rad-score. Radiomics features, gene mutation signatures, and clinical characteristics were first evaluated in univariate logistic regression analysis to determine whether they were candidate predictors for grade ≥ 2 RP. The confirmed related predictors were then included in multivariate logistic regression analysis to screen for independent risk factors. Finally, a comprehensive nomogram was established on the basis of multivariate analysis. The ROC curve and area under curve (AUC) were used to evaluate the predictive power of the model.

### Statistical analysis

LASSO regression analysis was performed using Python software (version 3.9, https://www.python.org/). The characteristics of patients in the RP and non-RP groups were compared using the chi-square test. Binary logistic regression analysis was used to determine independent predictors of RP using univariate and multivariate analyses. The DeLong test was used to analyze the statistical differences in the AUC values between the different models. Factors with a *p*-value < 0.10 in univariate analysis were included in the multivariate analysis. ROC curve analysis was conducted using MedCalc software (MedCalc Software Ltd.). The nomogram, calibration curve, and decision curve were calculated using R software (R Foundation for Statistical Computing). All statistical analyses were conducted using SPSS software (version 25.0; IBM Corp.). All tests were two-tailed, and *p* < 0.05 was considered to be statistically significant.

## Results

### Patient characteristics

We retrospectively analyzed 100 patients with unresectable stage III NSCLC who underwent dCRT. The main patient characteristics are summarized in Table 1. A total of 29 (29%) developed grade ≥2 RP in the whole cohort. The median interval from RT completion to grade ≥ 2 RP occurrence was 54 days. There was a difference in histology between the RP and non-RP groups; however, this difference was not statistically significant (*p* = 0.073). Other baseline data showed no significant differences between the two groups (Table 1).

**Table 1:** Comparison of patients’ characteristics between the non-RP and RPgroups

Variables		non-RP group (*N* = 71)	RP group (*N* = 29)	*p*
Gender	Male	60(84.5)	27(93.1)	0.405
	Female	11(15.5)	2(6.9)	
Age	≤ 60	33(46.5)	13(44.8)	0.880
	>60	38(53.5)	16(55.2)	
Smoking status	Never	18(25.4)	7(24.1)	0.899
	Former/current	53(74.6)	22(75.9)	
Tumor location	Right Lung	38(53.5)	16(55.2)	1.000
	Left Lung	31(43.7)	12(41.4)	
	Others	2(2.8)	1(3.4)	
Histology	ADC	28(39.4)	6(20.7)	0.073
	SCC	43(60.6)	23(79.3)	
Clinical stage	IIIA	32(45.1)	10(34.5)	0.260
	IIIB	34(47.9)	14(48.3)	
	IIIC	5(7.0)	5(17.2)	
Radiation dose	≥60 Gy	57(80.3)	21(72.4)	0.389
	≤ 60 Gy	14(19.7)	8(27.6)	
Chemoradiotherapy	SCRT	30(42.3)	15(51.7)	0.388
	CCRT	41(57.7)	14(48.3)	
Radiation therapy	3D-CRT	15(21.1)	9(31.0)	0.292
	IMRT	56(78.9)	20(69.0)	

Abbreviations: RP: radiation pneumonitis, ADC: adenocarcinoma, SCC: squamous cell carcinoma, SCRT: sequential chemoradiotherapy, CCRT: concurrent chemoradiotherapy, IMRT: intensity modulated radiation therapy, 3D-CRT: 3-dimensional conformal radiation therapy.

### Radiomics feature selection and signature construction

**Fig. 2:**
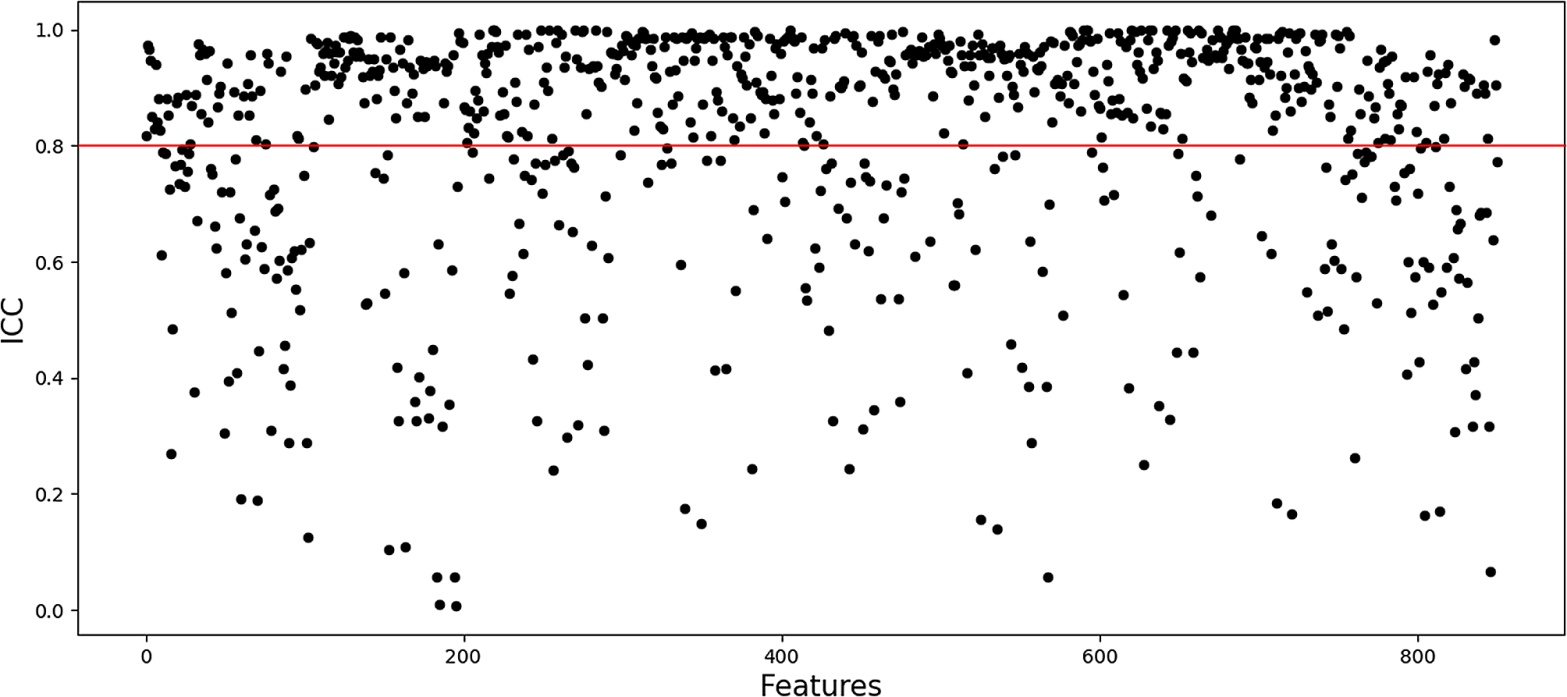
Evaluation of the stability of radiomics features based on the ICC. Features with an ICC of < 0.8 were removed, and the remaining 587 radiomics features were included in the subsequent data analysis as stable features. ICC: intraclass correlation coefficient

**Fig. 3:**
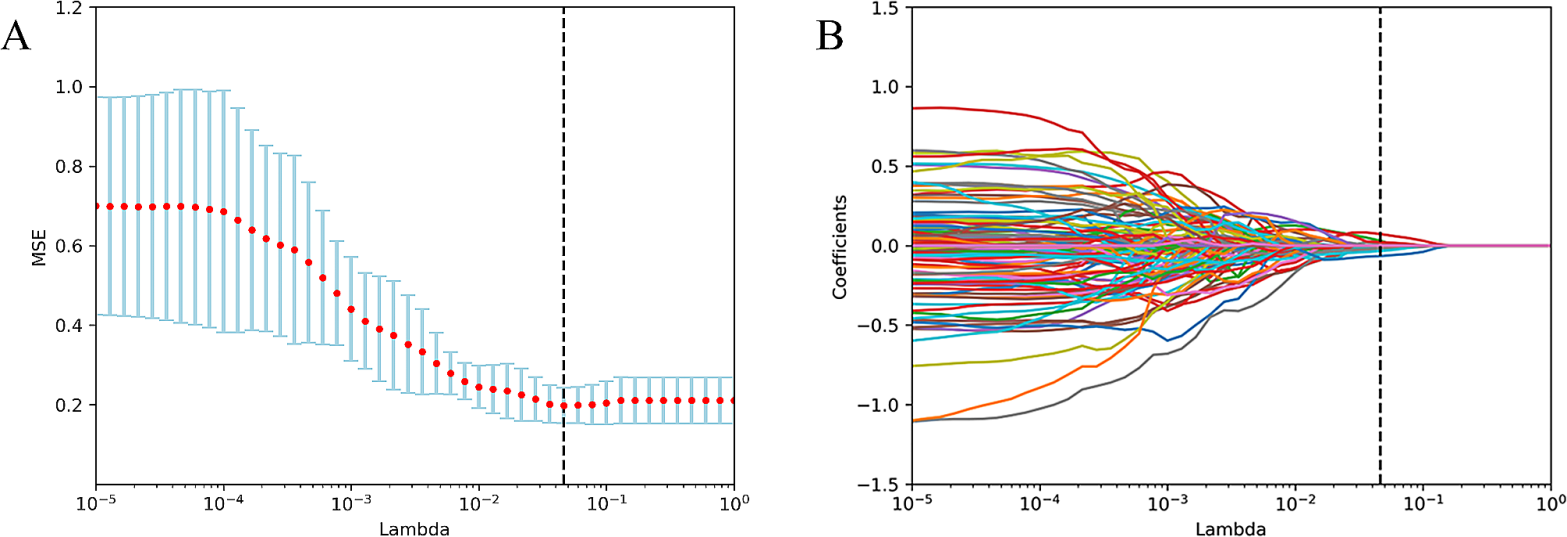
Selection of radiomic features associated with grade ≥ 2 RP based on the LASSO regression models. **A**: Cross-validation curve. The vertical axis represents the mean square error, whereas the horizontal axis represents the lambda (λ). **B**: Coefficient curves for radiomic features. The vertical axis represents the radiomic features’ coefficients, whereas the horizontal axis represents the λ. Nine features with non-zero coefficients were finally selected. RP: radiation pneumonitis, LASSO: least absolute shrinkage and selection operator

A total of 851 features from each patient’s ROI were extracted using the radiomics plug-in of 3D Slicer, including 18 first-order statistic features, 14 shape features, 75 texture features (24 Gy-level co-occurrence matrixes, 14 Gy-level dependence matrixes, 16 Gy-level run-length matrixes, 16 Gy-level size zone matrixes, 5 neighbor gray-tone difference matrixes), and 744 wavelet features. After excluding 264 ineligible features (features with an ICC of <0.8) (Figs. 2), 587 features were included in the subsequent data analysis as stable feature parameters. After LASSO regression analysis, nine radiomics features with non-zero coefficients were screened to develop a radiomics signature Rad-score (Fig. 3A and B). Subsequently, a fitting formula for the Rad-score was constructed on the basis of a linear combination of these selected features and the corresponding non-zero coefficients (Table 2). The calculation method of the Rad-score is as follows:

Rad-score.

= 0.0016216758231236334 × original_shape_LeastAxisLength.

+ -3.6599439786952596e-05 × wavelet-LLH_firstorder_Maximum.

+ -0.736174516564197 × wavelet-LLH_glcm_Idn.

+ 383.77372915203637 × wavelet-LHL_gldm_SmallDependenceLowGrayLevelEmphasis.

+ 62.47884571019595 × wavelet-LHL_glszm_SmallAreaLowGrayLevelEmphasis.

+ 0.04183439614802361 × wavelet-HLL_firstorder_Mean.

+ -0.39594655630028 × wavelet-HHH_firstorder_Skewness.

+ -78.25411961240329 × wavelet-LLL_glcm_Idmn.

**Fig. 4:**
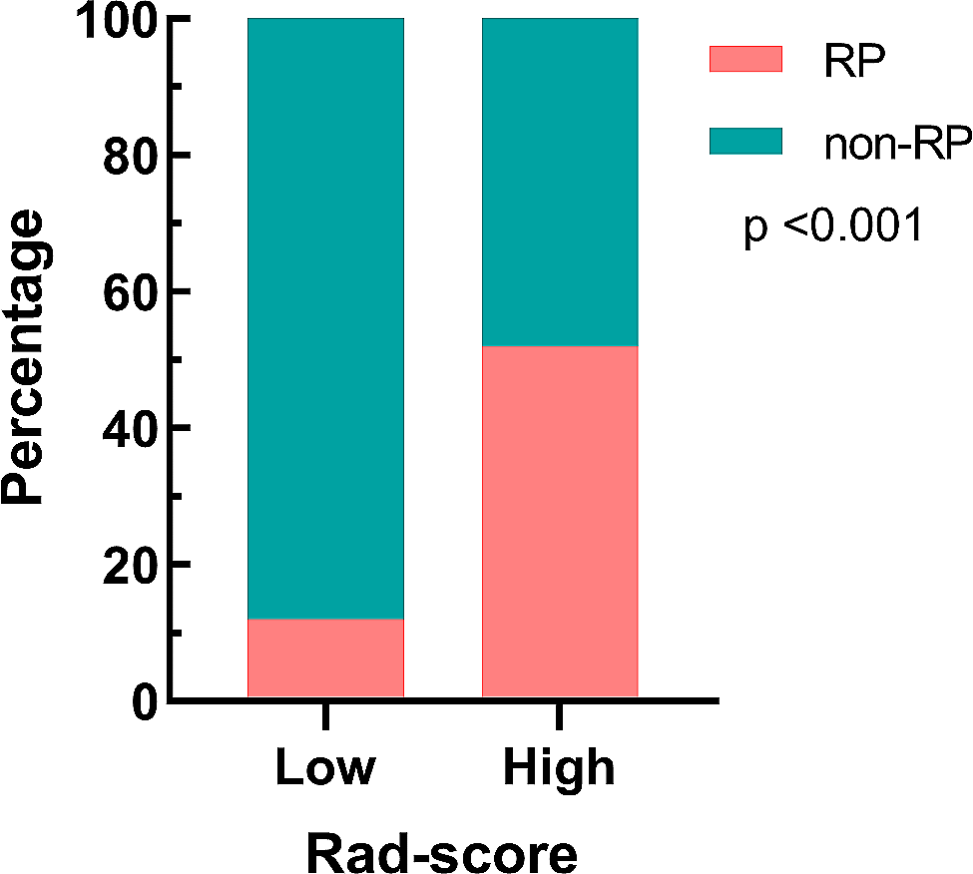
Difference in the incidence of grade ≥ 2 RP between the high and low Rad-score groups. Forty-two patients were divided into the high Rad-score group, and 22 of them developed grade ≥ 2 RP. Fifty-eight patients were divided into the low Rad-score group, and 7 of them developed grade ≥ 2 RP, respectively. There is a significant difference in the incidence of grade ≥ 2 RP between the two groups (52.4% vs. 12.1%, *p* < 0.001). RP: radiation pneumonitis, vs.: versus

The optimal cutoff value of the Rad-score was 0.32, and patients were divided into two groups with high and low Rad-scores. As shown in Fig. 4, the incidence of grade ≥ 2 RP was 52.4% (22/42) in the high Rad-score group compared with 12.1% (7/58) in the low Rad-score group (*p* < 0.001).

**Table 2:** Radiomics features associated with grade ≥ 2 RP selected by LASSO regression analysis

Radiomics features	Coefficients
original_shape_LeastAxisLength	0.0016216758231236334
wavelet-LLH_firstorder_Maximum	-3.6599439786952596e-05
wavelet-LLH_glcm_Idn	-0.736174516564197
wavelet-LHL_gldm_SmallDependenceLowGrayLevelEmphasis	383.77372915203637
wavelet-LHL_glszm_SmallAreaLowGrayLevelEmphasis	62.47884571019595
wavelet-HLL_firstorder_Mean	0.04183439614802361
wavelet-HHH_firstorder_Skewness	-0.39594655630028
wavelet-LLL_glcm_Idmn	-78.25411961240329

Abbreviations: LASSO: least absolute shrinkage and selection operator, RP, radiation pneumonitis

### Radiomics nomogram construction

**Fig. 5:**
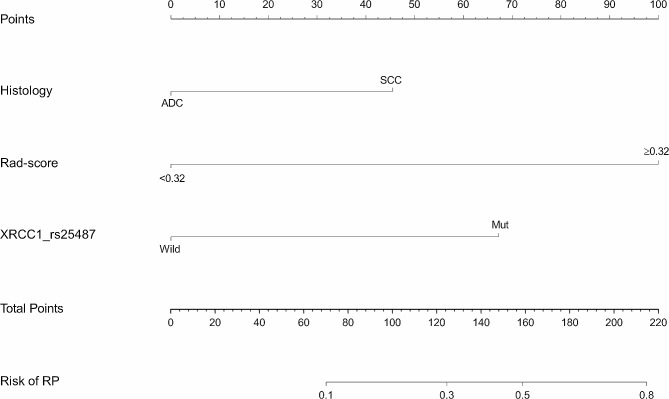
Nomogram incorporating histology, Rad-score, and XRCC1 (rs25487) allele mutation to predict grade ≥ 2 RP risk. ADC: adenocarcinoma, SCC: squamous cell carcinoma

**Fig. 6:**
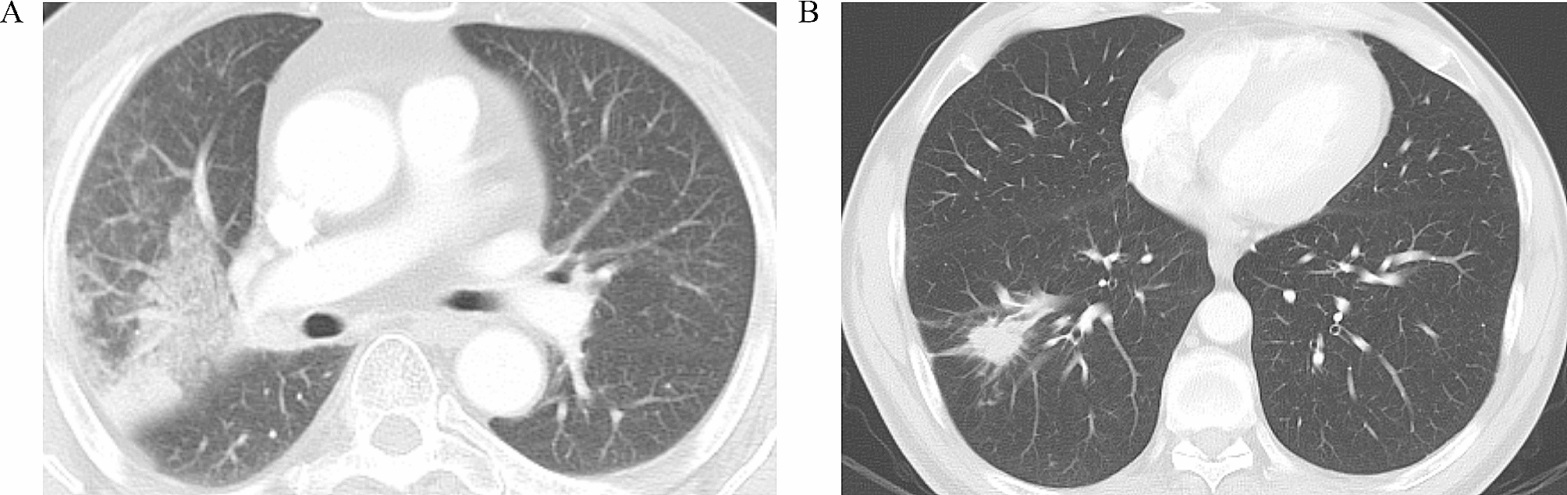
Representative images of patients with and without RP. (**A**) Grade 3 RP in an SCC patient with a high Rad-score and XRCC1 (rs25487) allele mutation. (**B**) Absence of RP in an ADC patient with a low Rad-score and wild-type XRCC1 (rs25487). RP: radiation pneumonitis, ADC: adenocarcinoma, SCC: squamous cell carcinoma

According to univariate analysis (Table 3), histology, Rad-score, and gene mutations in XRCC1 (rs25487) and NQO1 (rs1800566) were the potential high-risk factors that contributed to grade ≥ 2 RP development (all, *p* < 0.10). Multivariate analysis revealed that histology (*p* = 0.049), Rad-score (*p* < 0.001) and XRCC1 (rs25487) allele mutation (*p* = 0.004) were independent predictors for grade ≥ 2 RP (Table 3). On the basis of multivariate analysis, we developed a visible radiomics nomogram by combining histology, Rad-score, and XRCC1 (rs25487) allele mutation (Fig. 5). Typical representative images of patients with and without RP and corresponding high-risk factors are shown in Fig. 6.

**Fig. 7:**
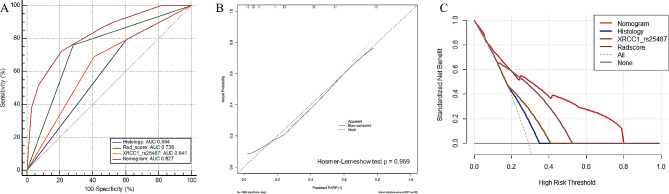
Predictive performance and calibration curve. (**A**) Predictive performance evaluation of each model for predicting grade ≥ 2 RP. The nomogram model shows superior prediction ability (AUC = 0.827) compared wtih the histology (AUC = 0.594), Rad-score (AUC = 0.738), and XRCC1 (rs25487) (AUC = 0.641) model. (**B**) Calibration curve of the nomogram model is presented as a solid line. The diagonal dashed line indicates perfect agreement. (**C**) Decision curves showing that the nomogram model to predict grade ≥ 2 RP probability has a greater benefit than any single model. RP: radiation pneumonitis, AUC: area under the curve

To test the predictive power of the nomogram model, ROC curves were constructed to compare the predictive performance of the nomogram and the other three independent predictors for RP. Based on the clinical factors, radiomics, and genomics models, the AUCs were 0.594, 0.738, and 0.641, respectively (Fig. 7A). The nomogram model obtained an AUC of 0.827 (Fig. 7A), which was significantly higher than those of histology (Delong test, *p* < 0.001), Rad-score (Delong test, *p* = 0.005), and XRCC1 (rs25487) (Delong test, *p* < 0.001). The consistency between the prediction of RP by the nomogram and actual observations was confirmed using calibration curves, and the Hosmer-Lemeshow test indicated no statistical difference between the predictive and actual values (*p* = 0.959). (Fig. 7B). The decision curves exhibited satisfactory positive benefits for the nomogram at the threshold probabilities (Fig. 7C).

**Table 3:** Results of univariate and multivariate analyses of grade ≥ 2 RP in the whole cohort

Variables		Univariate analysis		Multivariate analysis	
		OR (95% CI)	*p*	OR (95% CI)	*p*
Gender	Male	1			
	Female	0.404 (0.084–1.949)	0.259		
Age	≤ 60	1			
	>60	1.069 (0.449–2.546)	0.881		
Smoking status	Never	1			
	Former/current	1.067 (0.391–2.915)	0.899		
Tumor location	Left Lung	1	0.969		
	Right Lung	1.088 (0.448–2.638)	0.852		
	Others	1.292 (0.107–15.598)	0.840		
Histology	ADC	1			
	SCC	2.496 (0.903-6.900)	0.078	3.607 (1.006–12.937)	0.049
Clinical stage	IIIA	1	0.280		
	IIIB	1.318 (0.513–3.387)	0.567		
	IIIC	3.200 (0.767–13.353)	0.111		
Radiation dose	≥60 Gy	1			
	< 60 Gy	0.645 (0.237–1.757)	0.391		
Chemoradiotherapy	SCRT	1			
	CCRT	0.683 (0.287–1.626)	0.389		
Radiation therapy	IMRT	1			
	3D-CRT	1.680 (0.636–4.438)	0.295		
XRCC1 (rs25487)	Wild	1			
	Mutation	3.218 (1.285–8.060)	0.013	5.698 (1.749–18.561)	0.004
XRCC5 (rs3835)	Wild	1			
	Mutation	0.567 (1.148–2.181)	0.409		
MTHFR (rs1801133)	Wild	1			
	Mutation	2.451 (0.757–7.939)	0.135		
NQO1 (rs1800566)	Wild	1			
	Mutation	2.609 (0.886–7.679)	0.082	3.140 (0.842–11.716)	0.089
ZNF217	Wild	1			
	Amplification	2.556 (0.342–19.070)	0.360		
POLD1	Wild	1			
	Mutation	3.627 (0.758–17.355)	0.107		
Rad-score	Low	1			
	High	8.014 (2.962–21.685)	< 0.001	12.216 (3.693–40.410)	< 0.001

Abbreviations: ADC: adenocarcinoma, SCC: squamous cell carcinoma, SCRT: sequential chemoradiotherapy, CCRT: concurrent chemoradiotherapy, IMRT: intensity modulated radiation therapy, 3D-CRT: 3-dimensional conformal radiation therapy, CNV: copy number variation, OR: odds ratio, CI: confidence interval

## Discussion

In this study, we observed a strong quantitative relationship between CT image-based radiomics features and RP in patients with unresectable stage III NSCLC. Accordingly, the derived radiomics features proved to be promising CT-based biomarkers for predicting RP. We developed a nomogram model combining clinical factors, radiomics, and genomics on the basis of multivariate logistic regression analysis. More importantly, the combined model showed the best predictive ability compared with any the clinical factor, radiomics, or genomics model alone. This result showed that the combined nomogram model improved the ability of radiomics features and gene mutation signatures to predict the risk of RP development.

Radiomics is an emerging image analysis technique that can extract an amount of quantitative features from image data to quantify tumor heterogeneity, which is useful for personalized predictions [17, 18]. By providing a three-dimensional characterization of lesions, models based on radiomic features from CT have been developed to detect nodules [19], discriminate between malignant and benign lesions [20], and characterize histology [21], stage [22], and genotype [23]. Furthermore, radiomics has shown promising results in predicting radiation-induced lung injury [24]. Krafft et al. demonstrated that the addition of CT radiomics features extracted from the whole lung volume could improve prediction of RP in patients with NSCLC [25]. However, regarding the influence of endogenous factors and the radiosensitivity of lung tissue on RP occurrence [9], relying on standard clinical and radiomic features alone may not provide sufficient predictive accuracy. Thus, the present study established a combined model that incorporated clinical characteristics, radiomic features, and gene mutation signatures. Our research provides a new direction for individualized response-adapted decision-making for radiotherapy in NSCLC.

Gene-expression signatures, each composed of dozens to hundreds of genes, have the potential to improve diagnosis, prognosis, and prediction of treatment response [26]. Recently, the association between genetic factors and toxicity has been demonstrated in studies of genetic variants implicated in radiation-induced pneumonitis in patients with lung cancer [27]. These findings contribute to the identification of biological mechanisms and increase our understanding of the genetic factors that contribute to the susceptibility to radiation-induced adverse effects. To our best knowledge, several studies have suggested that the XRCC1 (rs25487) allele mutation serves as a potential biomarker for predicting RP in patients with NSCLC [28, 29]. However, these studies were based on traditional low-throughput sequencing methods. Taking advantage of the next-generation sequencing technology, our study provides further support for the association between the XRCC1 (rs25487) allele mutation and grade ≥ 2 RP.

In the era of modern personalized medicine, integrated multiomics approaches offer improved diagnostic accuracy and precise predictions. The integrated model combining radiomics with genomics outperformed either one alone in predicting prognosis or assessing postoperative recurrence risk in NSCLC [30, 31]. However, no previous studies have integrated radiomics and genomics to predict the risk of RP in patients with NSCLC. The present study aimed to fill this research gap and provide a unique perspective for identifying RP, which differs from conventional methods. Similarly, our combined model showed an optimal predictive performance. The current paradigm of gene expression profiling involves invasive surgery or biopsy procurement of tissue specimens. Unfortunately, this method presents considerable challenges including elevated costs, extended turnaround times, and technical complexity. These obstacles hamper the widespread implementation of gene expression profiling and limit its utility in a diverse range of patients with cancer. Radiogenomics, which highlights the link between radiomic features and gene expression patterns in patients with cancer, can be considered a substitute for genetic testing [32]. Thus, in future studies, we can investigate and establish correlations between low-cost and non-invasive image-based radiomic signatures and specific gene expression status in patients with RP.

Despite these findings, we acknowledge that our study had some limitations. This retrospective study had a small sample size, which may explain the low predictive accuracy of the model. A multi-center collaborative work was performed in our study to overcome this limitation, but external validation is lacking. The integrated prediction model developed in this study should be further validated using data from larger sample sizes.

## Conclusion

This study explored the utility of radiomics and genomics models as a feasible approach to predict grade ≥ 2 RP in patients with unresectable stage III NSCLC treated with dCRT. Compared with any clinical factor, radiomics model, or genomics model, the integrated model showed superior predictive performance. Our integrated model may be useful for early screening to identify patients wtih NSCLC who are predicted to be at a substantially greater risk of developing RP resulting from radiation exposure.

## Data Availability

The datasets used and analyzed during the current study are available from the corresponding author upon reasonable request.

## Abbreviations

NSCLC: Non–small cell lung cancer
RT: Radiotherapy
RILT: Radiation-induced lung toxicity
RP: Radiation pneumonitis
CT: Computed tomography
ROI: Region of interest
dCRT: Definitive chemoradiotherapy
ICC: Intraclass correlation coefficient
LASSO: Least absolute shrinkage and selection operator
ROC: Receiver operating characteristic
AUC: Area under the curve
ROI: Region of interest
